# Peripheral blood lymphocytes influence human papillomavirus infection and clearance: a retrospective cohort study

**DOI:** 10.1186/s12985-023-02039-6

**Published:** 2023-05-01

**Authors:** Ye Li, Yebin Feng, Yanlin Chen, Wenyu Lin, Hangjing Gao, Ming Chen, Kelvin Stefan Osafo, Xiaodan Mao, Yafang Kang, Leyi Huang, Dabin Liu, Shuxia Xu, Lixiang Huang, Binhua Dong, Pengming Sun

**Affiliations:** 1 Laboratory of Gynecologic Oncology, College of Clinical Medicine for Obstetrics & Gynecology and Pediatrics, Fujian Maternity and Child Health Hospital, Fujian Medical University, Fuzhou, Fujian 350001 China; 2 Fujian Key Laboratory of Women and Children’s Critical Diseases Research, Fujian Maternity and Child Health Hospital (Fujian Women and Children’s Hospital), Fuzhou, Fujian 350001 China; 3 Fujian Clinical Research Center for Gynecological Oncology, Fujian Maternity and Child Health Hospital (Fujian Obstetrics and Gynecology Hospital), Fuzhou, Fujian 350001 China; 4 Department of Scientific Research, Fujian Maternity and Child Health Hospital, Fuzhou, Fujian 350001 P.R. China; 5 Department of Gynecology, Fujian Maternity and Child Health Hospital, Fuzhou, Fujian 350001 P.R. China; 6 Department of Pathology, Fujian Maternity and Child Health Hospital, Fuzhou, Fujian 350001 P.R. China

**Keywords:** Human papillomavirus, peripheral lymphocyte subsets, HPV infection, HPV clearance, Prediction model

## Abstract

**Background:**

There is a close correlation between HPV infection and systemic immune status. The purpose of this study was to determine which lymphocytes in peripheral blood influence human papillomavirus (HPV) infection and to identify whether peripheral blood lymphocyte (PBL) subsets could be used as biomarkers to predict HPV clearance in the short term.

**Methods:**

This study involved 716 women undergoing colposcopy from 2019 to 2021. Logistic and Cox regression were used to analyze the association of PBLs with HPV infection and clearance. Using Cox regression, bidirectional stepwise regression and the Akaike information criterion (AIC), lymphocyte prediction models were developed, with the C-index assessing performance. ROC analysis determined optimal cutoff values, and their accuracy for HPV clearance risk stratification was evaluated via Kaplan‒Meier and time-dependent ROC. Bootstrap resampling validated the model and cutoff values.

**Results:**

Lower CD4 + T cells were associated with a higher risk of HPV, high-risk HPV, HPV18 and HPV52 infections, with corresponding ORs (95% CI) of 1.58 (1.16–2.15), 1.71 (1.23–2.36), 2.37 (1.12–5.02), and 3.67 (1.78–7.54), respectively. PBL subsets mainly affect the natural clearance of HPV, but their impact on postoperative HPV outcomes is not significant (P > 0.05). Lower T-cell and CD8 + T-cell counts, as well as a higher NK cell count, are unfavorable factors for natural HPV clearance (P < 0.05). The optimal cutoff values determined by the PBL prognostic model (T-cell percentage: 67.39%, NK cell percentage: 22.65%, CD8 + T-cell model risk score: 0.95) can effectively divide the population into high-risk and low-risk groups, accurately predicting the natural clearance of HPV. After internal validation with bootstrap resampling, the above conclusions still hold.

**Conclusions:**

CD4 + T cells were important determinants of HPV infection. T cells, NK cells, and CD8 + T cells can serve as potential biomarkers for predicting natural HPV clearance, which can aid in patient risk stratification, individualized treatment, and follow-up management.

**Supplementary Information:**

The online version contains supplementary material available at 10.1186/s12985-023-02039-6.

## Background

Human papillomavirus (HPV) is a small, nonenveloped, highly hoster-specific double-chain looped DNA virus [1]. To date, HPV has been identified as having more than 200 types [2]. HPV causes benign or malignant tumors by infecting mucous membranes and/or skin. Depending on its carcinogenic potential, HPV is classified as high-risk (HR-HPV) or low-risk (LR-HPV). Approximately 90% of cervical cancers worldwide are caused by HR-HPV, of which 70% are caused by HPV 16 and 18 infections [3]. Furthermore, HPV has been linked to anal and oropharyngeal cancer, genital warts and other skin diseases [2]. HPV infection is a public health problem.

Cervical epithelial cells are the primary targets of HPV infection. The HPV life cycle is closely related to epithelial differentiation, and no complete virus particles have been found in peripheral blood [2, 4]. Although HPV infection does not result in complete virus particles in peripheral blood, free HPV DNA or RNA fragments have been detected in the peripheral blood of patients with genitourinary infections, cervical cancer, oropharyngeal squamous cell carcinoma, head and neck cancer, and anal squamous cell carcinoma [2, 5, 6]. These fragments may originate from damaged cells or virus particles during the infection process. Additionally, HPV capsid components have also been reported to appear in peripheral blood [7]. These free virus nucleic acid fragments and capsid proteins. can act as exogenous antigens, stimulating the immune system and inducing a systemic immune response.

Although local immunity has been recognized as a key factor in clearing cervical HPV infection [8], it is essential to acknowledge that systemic immunity, including circulating immune cells and cytokines, may promote HPV infection clearance by interacting with local immune responses and providing a broader immunological background [9, 10]. Thus, understanding systemic immunity in the context of HPV infection and clearance may lead to more effective prevention and treatment strategies, so the importance of the role of systemic immunity should be emphasized.

The systemic immune status can be reflected by changes in peripheral blood lymphocyte (PBL) subsets. Current research on the impact of PBLs on HPV infection and clearance is limited, with only a few studies reporting changes in PBL subset counts due to HPV infection [11–14]. Spiwak et al. observed that women with HPV-induced cervical lesions had lower levels of peripheral blood CD4 + T cells and CD4/CD8 ratios, as well as increased CD8 + T-cell counts [13]. Lazarenko et al. found similar trends in the decrease in CD4 + T cells and CD4/CD8 ratios but no significant changes in CD8 + T-cell counts [14]. Although previous studies have demonstrated correlations between PBL subsets and HPV infection, they have not specified which HPV genotypes are susceptible to PBL fluctuations. Moreover, discussions on the impact of PBL subset changes on HPV clearance are scarce. Thus, the value of PBL subsets in HPV infection or clearance remains poorly elucidated.

We collected cross-sectional data from 716 women who underwent colposcopy and 12-month follow-up information from 164 HPV-positive patients. Our research objectives mainly include (1) investigating the relationship between PBLs and susceptibility to different HPV genotypes; (2) exploring whether PBLs affect the natural clearance of HPV in the short term; (3) examining whether PBLs influence postoperative HPV clearance in women with high-grade squamous intraepithelial lesions or worse (HSIL+); (4) identifying specific peripheral blood immune cells affecting HPV infection and clearance; and (5) establishing potential immunological marker thresholds to help more accurately predict HPV clearance. By investigating the above content, we aim to provide evidence-based references for rational patient risk stratification, individualized treatment, and follow-up.

## Materials and methods

### Study design

**Fig. 1:**
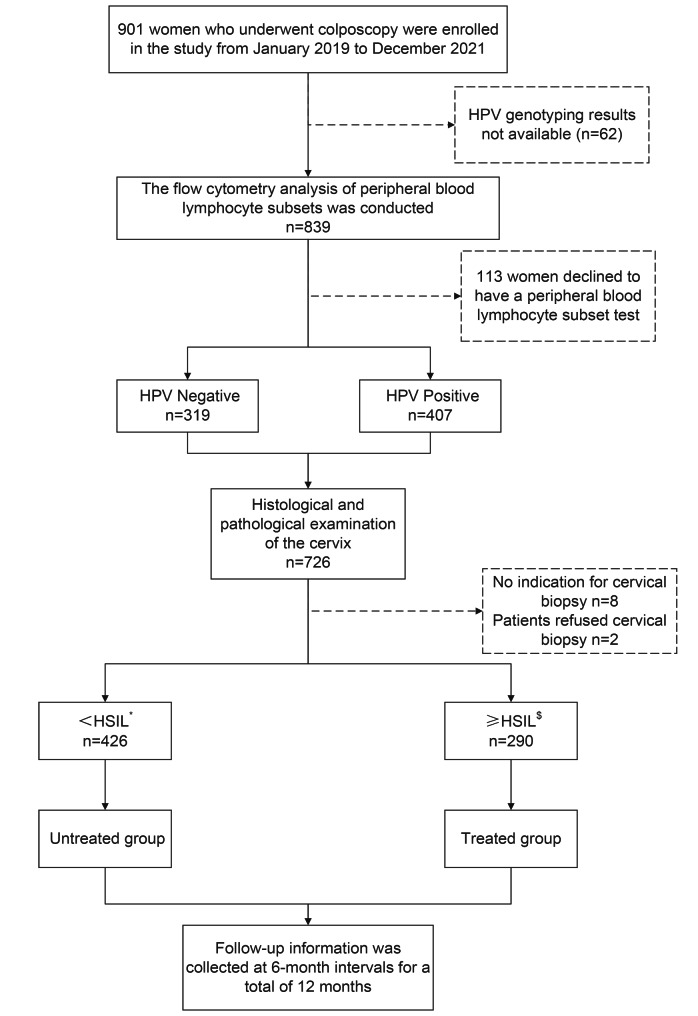
Study cohort flowchart. HSIL: high-grade squamous intraepithelial lesion. ^*^ Low-grade squamous intraepithelial lesion or no cervical lesion; ^$^ High-grade squamous intraepithelial lesion or worse

A retrospective cohort study evaluating the influence of PBL subsets on HPV infection as well as HPV clearance was conducted in Fuzhou, China. All subjects gave written informed consent to participate in the study. This study was approved by the Ethics Committee of Fujian Maternity and Child Health Hospital (2022KYLLR03050). The study population was recruited from women undergoing colposcopy in the Fujian Maternal and Child Health Hospital between January 2019 and December 2021. A total of 716 patients were enrolled. Inclusion criteria: Women who did not receive any dose of HPV vaccine; no condom use at least the past year; PBL subsets and cervical HPV genotyping were tested during the initial visit; clinical diagnoses were confirmed by pathology and immunohistochemical staining; Exclusion criteria: sexual partner greater than 1, history of smoking, abnormal immune function; abnormal white blood cell count and C-reactive protein, positive serological tests for hepatitis B, hepatitis C, syphilis, and HIV; presence of acute or chronic infection symptoms or signs; use of antibiotics, antiviral drugs, hormones, and immunotherapy drugs within the past month; having received or currently undergoing chemotherapy and radiation therapy; suffering from malignant tumors or serious heart, lung, liver, or kidney diseases. The correlation between PBL percentages and different types of HPV infection (mainly infection with a single HPV type) was analyzed. Follow-up information was collected (see Fig. 1) at 6-month intervals for a total of 12 months, with a focus on HPV regression. HPV clearance was defined as long as at least one HPV-negative event occurred during the follow-up period. HPV persistence was defined as consecutive positive HPV tests, regardless of genotype. Women with low-grade squamous intraepithelial lesions (LSILs) or no cervix lesions did not receive treatment. Women with HSIL+ underwent surgical treatment. Surgical modalities include cervical conization, hysterectomy, LEEP, and electrocautery of the cervix.

### HPV genotyping assay

The Human Papillomavirus Genotyping Kit for 23 Types (PCR-Reverse Dot Blot, Yaneng® Biosciences) was used to identify 23 genotypes in exfoliated cervical cells, including 14 high-risk HPV genotypes (HPV-16, -18, -31, -33, -35, -39, -45, -51, -52, -56, -58, -59, and − 68), 4 possible high-risk HPV genotypes (HPV-53, -66, -73, and − 82), and 5 low-risk HPV genotypes (HPV-6, -11, -42, -43, -81, and − 83). Briefly, the extracted HPV DNA in a 25 µl reaction system was amplified with the PGMY09/PGMY11 primer, and 5 µL of the extracted HPV DNA served as a control. After amplification, the PCR products were immobilized onto a nitrocellulose membrane and hybridized with 23 different fixed type-specific probes. The results were recorded by directly visualizing blue spots on the membrane. All the detection procedures strictly followed the manufacturer’s instructions.

### Flow cytometric analysis of PBLs

Fresh peripheral blood samples (5 ml) were collected from patients using EDTA anticoagulant tubes. Twenty microliters of BD Multitest 6-color TBNK reagent (BD catalog number 337,166) was placed into the bottom of a BD Trucount tube, and 50 µl of heparinized whole blood was added to the tube, mixed with vortex, and incubated for 15 min protected from light. Then, 450 µl of 1X BD FACS lysing solution was added and incubated again for 15 min protected from light. Finally, with a BD Canto II, we analyzed fresh blood samples for total Lym subset distributions, including T lymphocytes, B lymphocytes, NK cells, CD4 + T lymphocytes, and CD8 + T lymphocytes. All manipulations were carried out at room temperature (20℃-25℃).

### Prognostic model construction

Univariate and multivariate analyses were performed using Cox proportional hazard regression to evaluate independent prognostic factors associated with HPV clearance under untreated and treated conditions. Bidirectional stepwise regression analysis and Akaike information criterion (AIC) were used to select the best variable combination for the lymphocyte model. Risk scores for each lymphocyte model were calculated based on the selected best variable combination using the formula Risk score = ho(t)* *exp*(β1 × 1 + β2 × 2 + …+βnXn), where “X” represents the percentage of lymphocytes, β represents the coefficients of lymphocytes in the model, and ho(t) represents the baseline hazard function. The optimal cutoff point for each model’s risk score was determined using ROC analysis, dividing the risk scores into high-risk and low-risk groups. Combining the optimal cutoff value with the lymphocyte model to predict HPV clearance.

### Evaluation and validation of the prognostic model

The predictive accuracy of univariate and multivariate Cox regression models was evaluated using the concordance index (C-index). Visualize the impact of changes in the lymphocyte model risk scores on HPV clearance through risk score distribution, risk status, and risk heatmap. Kaplan‒Meier curves and log-rank tests were used to compare the differences in HPV clearance rates between the high-risk and low-risk groups defined by the optimal cutoff value. Time-dependent receiver operating characteristic (ROC) curves were used to evaluate the accuracy of the optimal cutoff value in predicting natural HPV clearance. Subsequently, internal validation of the lymphocyte prognostic model was performed using bootstrap resampling (with 1,000 bootstrap samples). Bootstrap resampling is specifically applied to the following steps: determination of prognostic factors related to HPV clearance in univariate and multivariate Cox regression analyses, evaluation of the predictive performance of Cox models using the C-index, employment of bidirectional stepwise regression analysis and AIC method to select the best variable combination, validation of the generalizability of the optimal cutoff point, and evaluation of the accuracy of the optimal cutoff point in predicting natural HPV clearance using time-dependent ROC.

### Statistical analysis

The Kolmogorov‒Smirnov test was used to assess whether the data followed a normal distribution. Independent sample t tests or Mann‒Whitney U tests were used to compare differences between two groups of continuous variables. Chi-square tests or Fisher’s exact tests were employed for comparisons of categorical variables between groups. Lymphocyte subsets were dichotomized using the median value. Chi-square tests were used to evaluate differences in lymphocyte subsets between the HPV-positive and HPV-negative groups. Binary logistic regression was utilized to assess the relationship between PBL subsets and different HPV genotypes (adjusted for age and gravidity). The above statistical analysis was performed by IBM SPSS Statistics 25. Univariate and multivariate Cox regression analyses, bidirectional stepwise regression, AIC calculation, risk score calculation, and log-rank tests were all performed using the survival package. The optimal cutoff value for the risk score was determined using the ggrisk package. Time-dependent ROC curves were plotted using the risksetROC package, and the area under the curve (AUC) was calculated for each curve. Bootstrap resampling was used for internal validation of the results of univariate and multivariate Cox regression analyses, bidirectional stepwise regression, AIC calculation, and the generalization ability of the optimal cutoff value. The boot package was used for bootstrap resampling. The plots were generated using the ggplot2 package and Hiplot (ORG) platform (https://hiplot.cn/). These statistical analyses were performed using R software (version 4.1.3).

## Results

### Patient characteristics and results

A total of 901 women who underwent colposcopy at initial treatment were recruited. A total of 185 women who did not meet the inclusion criteria were excluded. A total of 716 women were included in the final analysis. Among them, 309 women had no HPV infection, 407 women had HPV infection, 341 women had HR-HPV infection only, 106 women had HPV 16 infection only, 33 women had HPV 18 infection only, 46 women had HPV 52 infection only, 23 women had HPV 56 infection only, 34 women had HPV 58 infection only, and 54 women had other HR-HPV infections (including HPV-31, -33, -35, -39, -45, -51, -59, -66, -68). Follow-up information was collected for 407 HPV-infected women. A total of 243 women lacking follow-up information were excluded. Table S7 shows the baseline characteristics of patients involved in follow-up and those not involved. A total of 164 women were included in the final analysis, of whom 103 received surgical treatment and 61 did not (Fig. 1). By 12 months, 106 women had HPV clearance, 78 (75.73%) in the treated group and 28 (45.90%) in the untreated group. 22 women had HPV persistence by 12 months, 14 cases (13.59%) in the treated group and 8 cases (13.12%) in the untreated group. Thirty-six women were lost to follow-up before HPV clearance, 11 (10.68%) in the treated group and 25 (40.98%) in the untreated group, and were classified as “HPV persistence” at 12 months.

Patient characteristics at baseline are detailed in Table 1. More than half of the HPV-infected women were younger than 46 years (234/406, 57.64%), had fewer than 3 pregnancies (248/405, 61.23%) and were HSIL+ (261/407, 64.13%). Nearly three-quarters of HPV-infected women were menopausal at enrollment (295/407, 72.48%) and had no more than 2 deliveries (306/406, 75.37%). For the HPV-negative population, more than half of the women were older than 46 years (168/309, 54.37%), approximately three-quarters had no more than 3 pregnancies (240/309, 77.67%), no more than 2 deliveries (248/309, 80.26%) and were menopausal at enrollment (229/309, 74.11%). Almost all HPV-negative women had no cervical lesions or LSIL only (280/309, 90.61%). Between the HPV-negative and HPV-positive groups, age, gravidity, and cervical lesions differed significantly (P < 0.05), while there were no statistically significant differences in parity and menopause (Table 1).

**Table 1:** Basic characteristics of the included patients

Characteristics	HPV Negative n = 309 (%)	HPV Positive n = 407 (%)	All n(%)	*P*
Age in years				
≤ 46	141/309(45.63)	234/406 (57.64)	375/715(52.45)	<0.01*
>46	168/309(54.37)	172/406 (42.36)	340/715(47.55)	
Gravidity				
≤ 3	240/309(77.67)	248/405(61.23)	488/714(68.35)	<0.01*
>3	69/309 (22.33)	157/405 (38.77)	226/714(31.65)	
Parity				
≤ 2	248/309 (80.26)	306/406 (75.37)	554/715(77.48)	0.12
>2	61/309 (19.74)	100/406 (24.63)	161/715(22.52)	
Menopause				
Yes	229/309 (74.11)	295/407(72.48)	524/716(73.18)	0.63
No	80/309 (25.89)	112/407(27.52)	192/716(26.82)	
Cervical Lesions				
Normal/LSIL	280/309 (90.61)	146/407(35.87)	426/716(59.50)	<0.01*
HSIL+	29/309 (9.39)	261/407(64.13)	290/716(40.50)	

* Calculated by chi-square test statistically significant at p < 0.05

HSIL+, high-grade squamous intraepithelial lesion or worse;

LSIL, low-grade squamous intraepithelial lesion

### Different types of HPV infection are affected by changes in PBL subsets

**Fig. 2:**
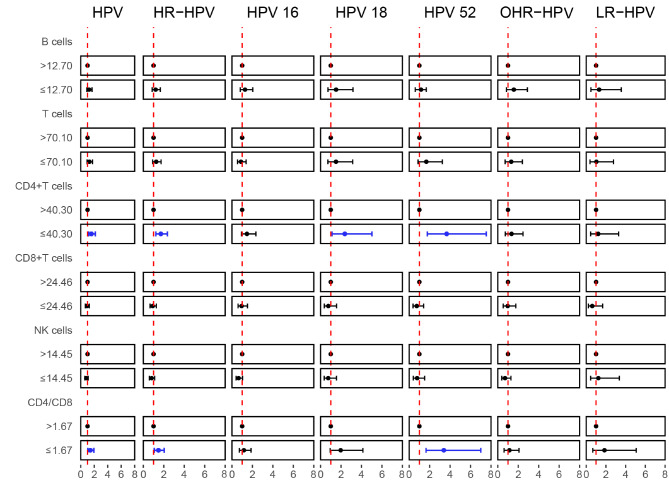
The association between peripheral blood lymphocyte subset percentages and different types of HPV infection (VS uninfected) at baseline. The percentages of B cells, T cells, CD4 + T cells, CD8 + T cells and NK cells as well as the CD4/CD8 ratio were dichotomized according to the median value. Odds ratios were adjusted for age and gravidity. The blue lines indicate that the difference was meaningful (P < 0.05), while the black lines indicate that the difference was not significant (P > 0.05). Low CD4 + T-cell percentages often facilitate HPV (OR 1.58, 95% CI 1.16–2.15, P<0.05), HR-HPV (OR 1.71, 95% CI 1.23–2.36, P<0.05), HPV18 (OR 2.37, 95% CI 1.12–5.02, P<0.05) and HPV52 infections (OR 3.67, 95% CI 1.78–7.54, P<0.05), while low CD4/CD8 ratios facilitate HPV (OR 1.42, 95% CI 1.04–1.94, P<0.05), HR-HPV (OR 1.48, 95% CI 1.07–2.05, P<0.05) and HPV52 infections (OR 3.39, 95% CI 1.65-7.00, P<0.05). HR-HPV: High-risk HPV including HPV-16, -18, -31, -33, -35, -39, -45, -51, -52, -56, -58, -59, -66, -68; LR-HPV: Low-risk HPV including HPV-6, -11, -42, -43, -81; OHR-HPV: Other high-risk HPV including HPV-31, -33, -35, -39, -45, -51, -59, -66, -68; OR: Odd Ratio

There were significant differences in PBL subsets between HPV-infected and uninfected women, which mainly reflected changes in the CD4 + T-lymph percentage and CD4/CD8 ratio. A decreased percentage of CD4 + T cells was associated with increased HPV, HR-HPV, HPV18, and HPV52 infections (Table S1, P < 0.05). A decreased CD4/CD8 ratio was also associated with increased HPV, HR-HPV, and HPV52 infections (Table S1, P < 0.05). The PBLs were divided into low expression and high expression groups using the median as the cutoff value. The results of the multivariate analyses are shown in Fig. 2. Compared to the high expression group, women with low expression of CD4 + T cells had an increased HPV infection rate by 0.58 times (OR 1.58, 95% CI 1.16–2.15), HR-HPV infection rate by 0.71 times (OR 1.71, 95% CI 1.23–2.36), HPV18 infection rate by 1.37 times (OR 2.37, 95% CI 1.12–5.02) and HPV52 infection rate by 2.67 times (OR 3.67, 95% CI 1.78–7.54). Compared with the group with a higher ratio, women with a lower CD4/CD8 ratio had an increased HPV infection rate by 0.42 times (OR 1.42, 95% CI 1.04–1.94), HR-HPV infection rate by 0.48 times (OR 1.48, 95% CI 1.07–2.05) and HPV52 infection rate by 2.39 times (OR 3.39, 95% CI 1.65-7.00). Univariate analysis showed that the percentage of B lymphocytes was different between other HR-HPV-positive and HPV-negative patients (Table S1, P < 0.05), but the difference was not statistically significant after multivariate adjustment (Fig. 2, P > 0.05). T cells, CD8 + T cells and NK cells in peripheral blood do not seem to affect the infection rate of HPV.

### Screening PBLs that affect HPV clearance

In the untreated population, T cells (HR (95% CI) vs. Bootstrap HR (95% CI): 1.09 (1.01–1.16) vs. 1.09 (1.01–1.18), C-index vs. Bootstrap C-index: 0.57 vs. 0.69) and NK cells (HR (95% CI) vs. Bootstrap HR (95% CI): 0.89 (0.83–0.97) vs. 0.88 (0.81–0.97), C-index vs. Bootstrap C-index: 0.68 vs. 0.72) were found to have significant prognostic value for predicting natural HPV clearance (Table 2 and S2). After adjusting for confounding factors (age, gravidity, parity, and menopausal status), CD8 + T cells, which were not significant in univariate analysis, were also considered a good predictor for natural HPV clearance (HR (95% CI) vs. Bootstrap HR (95% CI): 1.08 (1.01–1.16) vs. 1.09 (1.01–1.18), C-index vs. Bootstrap C-index: 0.63 vs. 0.69, Table 2 and S2). The C-index generally increased after bootstrap resampling, reflecting the accuracy of PBL in predicting HPV clearance (Tables S2). However, no PBL subsets associated with HPV clearance were identified in the treated population (P > 0.05; Table 3 and S3). These findings suggest that peripheral blood T cells, NK cells, and CD8 + T cells are closely associated with natural HPV clearance and can serve as biomarkers for predicting HPV outcomes.

**Table 2:** Effects of different variables on natural clearance of HPV infection

Characteristics	HPV Clearance _(Univariate)_					HPV Clearance _(Multivariate)_			
	P_(Unadjusted)_	HR_(Unadjusted)_	95%CI	C-index		P_(Adjusted)_	HR_(Adjusted)_	95%CI	C-index
Age in years	0.97	1.00	0.96–1.04	0.43					
Gravidity	0.48	1.11	0.83–1.47	0.54					
Parity	0.43	0.84	0.54–1.30	0.57					
Menopause									
No									
Yes	0.91	1.06	0.36–3.10	0.51					
B Cells	0.46	1.04	0.94–1.15	0.58		0.40	1.05	0.94–1.16	0.59
T Cells	0.01*	1.08	1.02–1.15	0.59		0.02*	1.09	1.01–1.16	0.57
CD4 + T Cells	0.27	1.04	0.97–1.11	0.56		0.36	1.03	0.96–1.11	0.61
CD8 + T Cells	0.10	1.05	0.99–1.11	0.57		0.03*	1.08	1.01–1.16	0.63
NK Cells	0.01*	0.91	0.85–0.98	0.64		<0.01*	0.89	0.83–0.97	0.68
CD4/CD8	0.51	0.77	0.36–1.67	0.54		0.31	0.63	0.26–1.53	0.56

Calculated by univariate and multivariate Cox regression

Multivariate Cox regression analysis was adjusted for age, gravidity, parity, and menopause

* Statistically significant at p < 0.05

C-index: concordance index

HR: hazard ratio

CI: confidence interval

**Table 3:** Effect of different variables on clearance of HPV infection after treatment in women with HSIL or worse lesions

variables	HPV Clearance _(Univariate)_					HPV Clearance _(Multivariate)_			
	P_(Unadjusted)_	HR_(Unadjusted)_	95%CI	C-index		P_(Adjusted)_	HR_(Adjusted)_	95%CI	C-index
Age in years	0.02*	0.98	0.96-1.00	0.54					
Gravidity	0.60	0.96	0.83–1.11	0.51					
Parity	0.04*	0.81	0.66–0.99	0.57					
Menopause									
No									
Yes	0.04*	0.58	0.34–0.98	0.55					
B Cells	0.09	1.04	0.99–1.09	0.56		0.07	1.04	1.00-1.09	0.62
T Cells	0.41	0.99	0.96–1.02	0.51		0.12	0.98	0.95–1.01	0.59
CD4 + T Cells	0.42	0.98	0.95–1.02	0.52		0.82	1.01	0.96–1.05	0.59
CD8 + T Cells	0.53	0.99	0.96–1.02	0.48		0.09	0.97	0.95-1.00	0.60
NK Cells	0.82	1.00	0.97–1.03	0.52		0.63	1.01	0.98–1.04	0.58
CD4/CD8	0.49	0.90	0.67–1.21	0.52		0.63	1.09	0.77–1.54	0.58

Calculated by univariate and multivariate Cox regression

Multivariate Cox regression analysis was adjusted for age, gravidity, parity, and menopause

C-index: concordance index

HSIL: high-grade squamous intraepithelial lesion

* Statistically significant at p < 0.05

HR: hazard ratio

CI: confidence interval

### Selecting the optimal variable combination for the lymphocyte prognostic model

**Fig. 3:**
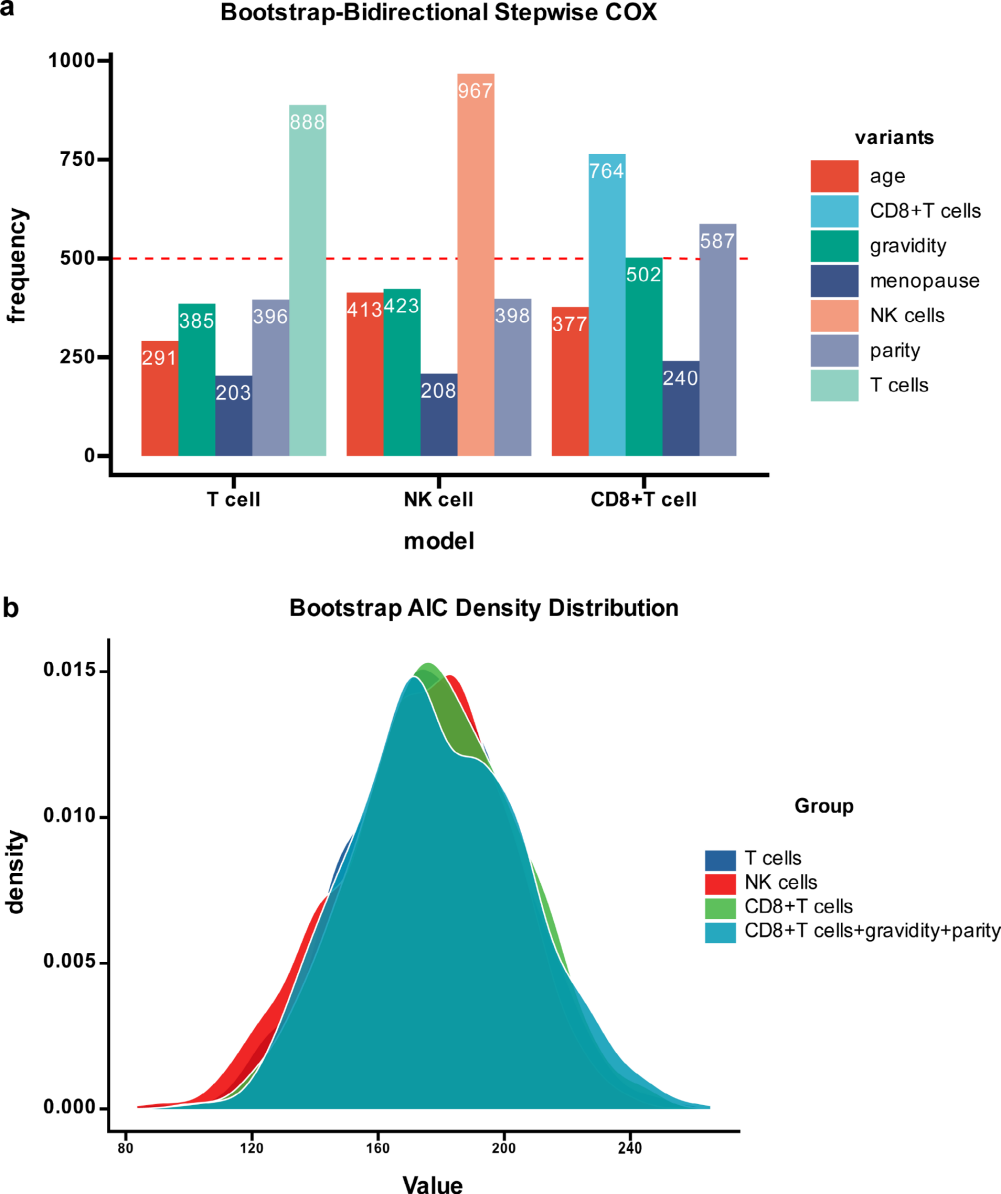
Identification of the optimal variable combination for peripheral blood lymphocyte prognostic models. a represents the result plot of the bidirectional stepwise regression bootstrap resampling. The vertical axis represents the frequency of each independent variable being drawn in the 1000 resampling processes, while the horizontal axis represents three different lymphocyte prediction models. Each subgroup in the models represents the total independent variables initially included in the analysis. The red dashed line at X = 500 indicates the threshold, and independent variables with a frequency greater than 500 will be included in their respective models. From a, it can be inferred that T cells, NK cells, and the combination of CD8 + T cells, gravidity and parity are the best independent variable combinations for the T-cell model, NK cell model, and CD8 + T-cell model, respectively. The distribution plot of the AIC values after bootstrap resampling for the best variable combination represented by b with the lowest/second lowest AIC. The sampling distribution of AIC values for the four independent variable combinations in the plot is relatively tight and concentrated, indicating that the represented lymphocyte prognosis model is stable and reliable

The results of bidirectional stepwise regression analysis indicate that T cells and NK cells can independently predict HPV clearance, while CD8 + T cells need adjustment for gravidity and parity to predict HPV clearance. These findings remain valid after internal validation using bootstrap resampling (with 1,000 samples) (Fig. 3a). In addition, the AIC values for possible combinations of lymphocytes and confounding factors showed that individual T cells (177.05), NK cells (175.33), and CD8 + T cells (180.98) had the lowest values. Since the univariate Cox regression of CD8 + T cells for predicting natural clearance of HPV was not statistically significant, the combination of CD8 + T cells, gravidity and parity with the second lowest AIC value (181.46) was considered the optimal combination, consistent with the results of bidirectional stepwise regression. Fig. 3b shows that after bootstrap resampling, the AIC distribution trend of the optimal variable combinations based on the lowest/second lowest AIC values was relatively concentrated, indicating that the selected lymphocyte prognostic models have stability and reliability.

### Determine the optimal cutoff values and preliminarily evaluate their predictive performance

**Fig. 4:**
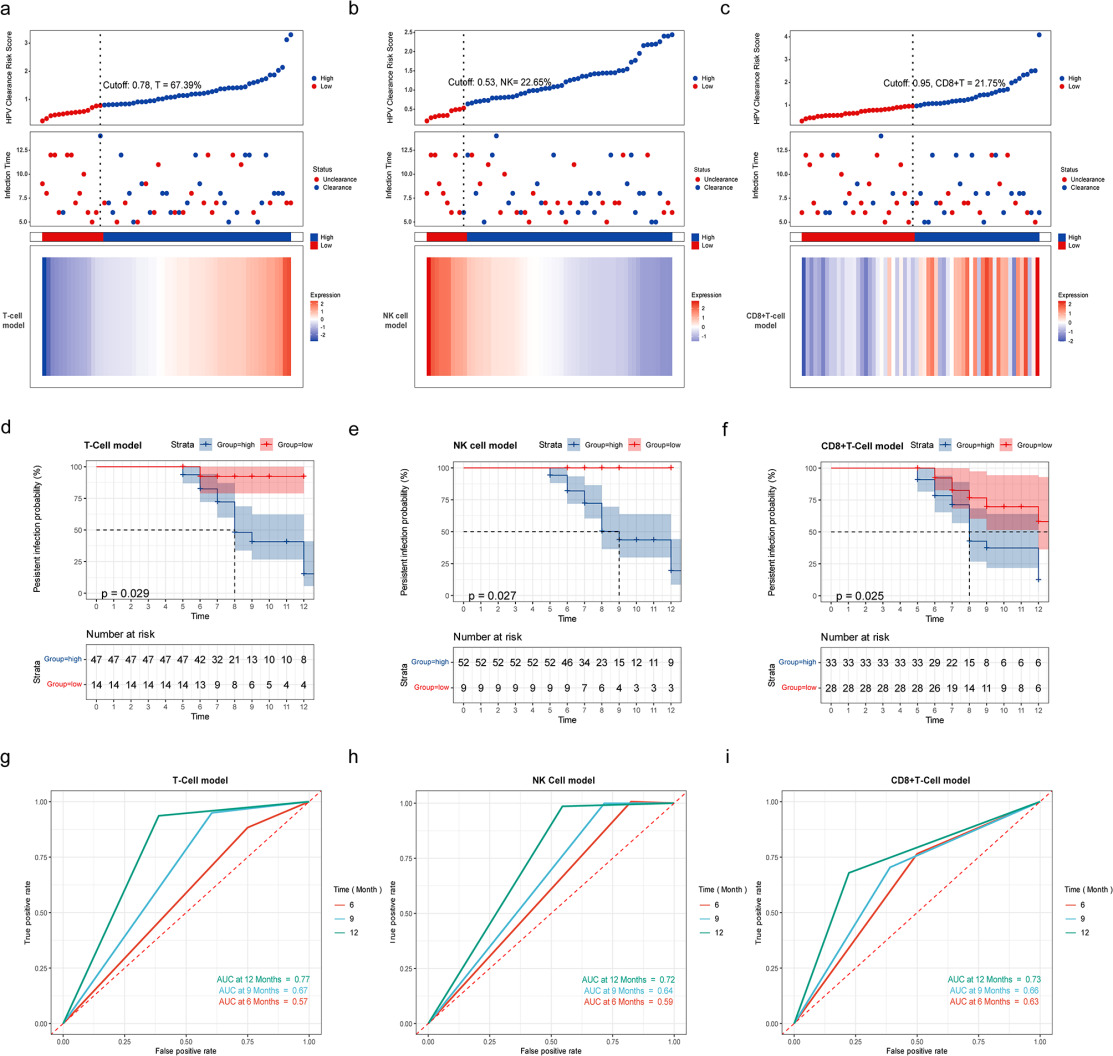
The performance of the optimal cutoff values determined by the T-cell model, NK cell model, and CD8 + T-cell model in predicting natural HPV clearance. We calculated the risk scores for predicting natural HPV clearance using the T-cell model, NK cell model, and CD8 + T-cell model (a-c) and plotted risk factor plots based on these risk scores. These plots include risk score ranking (top), HPV infection status (middle), and heatmaps of T-cell percentage, NK cell percentage, and CD8 + T-cell percentage (bottom). The optimal cutoff values determined by ROC analysis divided patients into high-risk and low-risk groups for natural HPV clearance. Kaplan‒Meier curves and log-rank tests were used to analyze the differences in natural HPV clearance between the high-risk and low-risk groups (d represents the T-cell model, e represents the NK cell model, f represents the CD8 + T-cell model). Time-dependent ROC curves were used to evaluate the accuracy of binary classification based on the optimal cutoff values in predicting HPV clearance (g represents T-cell model, h represents NK cell model, i represents CD8 + T-cell model). ROC, receiver operating characteristic; AUC, area under the curve; CI, confidence interval; HR, hazard ratio

Risk scores were calculated for three models (univariate T cell, univariate NK cell, and multivariate CD8 + T cell), and optimal cutoff values for risk scores were determined using ROC analysis (T: 0.78, NK: 0.53, CD8 + T: 0.95) to distinguish high-risk and low-risk patients. A higher risk score indicates a higher likelihood of natural HPV clearance. In the univariate models, the percentage of T cells or NK cells was linearly related to the risk score (Fig. 4a-c), and the corresponding lymphocyte percentages (T: 67.39%, NK: 22.65%) can be used for classification. Significant differences in HPV clearance outcomes between the high-risk and low-risk groups were observed in all three models (P < 0.05, Fig. 4d-f). Using the optimal cutoff values for classification provided relatively high accuracy in predicting natural HPV clearance within 12 months (Fig. 4g-i). The corresponding AUC values for the three models were 0.77 (T-cell model), 0.72 (NK cell model), and 0.73 (CD8 + T-cell model).

### Evaluating the generalizability of the optimal cutoff value in internal validation

We classified each sample into high- and low-risk groups using the optimal cutoff values for T-cell percentage, NK cell percentage, and CD8 + T-cell model risk score and performed internal validation using 1000 bootstrap resamples. The results showed that accurate prediction of natural HPV clearance could still be achieved after bootstrap resampling by using the optimal cutoff values for T-cell percentage (67.39%) (Bootstrap HR (95% CI): 1.09 (1.01–1.14)), NK cell percentage (22.65%) (Bootstrap HR (95% CI): 0.91 (0.85–0.99)), and CD8 + T-cell model risk score (0.95) (Bootstrap HR (95% CI): 6.03(1.59–29.03)) as the threshold points (Table 4). The predictive accuracy remained high before and after bootstrap resampling for T-cell AUC (0.77 vs. 0.76), NK cell AUC (0.72 vs. 0.69), and CD8 + T-cell AUC (0.73 vs. 0.64) (Fig. 4g-i; Table 4).

**Table 4:** Validation of the optimal cutoff value generalizability using bootstrap resampling

Cutoff values	Bootstrap HR (95%CI)	C-index	Bootstrap C-index	Bootstrap AUC
T cells^p^ (%)				
<67.39	1			
≥ 67.39	1.09(1.01–1.14) *	0.61	0.61	0.76
NK cells^p^ (%)				
<22.65	1			
≥ 22.65	0.91(0.85–0.99) *	0.60	0.60	0.69
CD8 + T cells Model RS				
<0.95	1			
≥ 0.95	6.03(1.59–29.03) *	0.63	0.68	0.64

Calculated by univariate Cox regression, receiver operating characteristic analysis and bootstrapping

Bootstrap HR (95% CI): average hazard ratio across 1,000 iterations and average 95% confidence interval for hazard ratio across 1,000 iterations

C-index: concordance index of the original data Cox model

Bootstrap C-index: average concordance index across 1,000 iterations

Bootstrap AUC: average area under the curve across 1,000 iterations

* Statistically significant variables: Variables with a mean hazard ratio (HR) and corresponding mean 95% confidence interval (CI) that are entirely between 0 and 1 or greater than 1

^p^ Lymphocyte percentage value

RS: Risk score, CD8 + T-cell model risk score

## Discussion

Our study highlights the crucial role of PBL subsets in HPV infection and clearance. We found that changes in the composition of these lymphocyte subsets may influence an individual’s susceptibility to HPV infection and their ability to effectively clear the virus.

By comparing the percentage of PBLs in HPV-infected versus HPV-uninfected women, we were surprised to find that the low CD4 + T-cell percentage was an important determinant of HPV infection. A lower CD4 cell percentage often promoted higher infection rates of HR-HPV, HPV18 and HPV52. In addition, the high prevalence of HPV is also related to a decrease in the CD4/CD8 ratio, which mainly increases the incidence of HR-HPV and HPV52 infection.

CD4 + T cells are an integral part of successful cellular and humoral host immune defense. The activity of CD4 cells has a direct influence on the function and superiority of B cells and T cells [15]. The CD4/CD8 ratio can reflect the status of cellular immunity and immune responses of the body to a certain degree. At baseline, a lower percentage of CD4 + T cells and a reduced CD4/CD8 ratio indicate a weak systemic immune status [16]. When the body is in an immunosuppressed state, the chance of HPV infection is greatly increased.

Previous studies have reported that women with lower CD4 + T-cell counts have a higher rate of HR-HPV infection and a higher HPV load [17, 18]. Women infected with HPV18 had defective HPV-18 E6 and E7 CD4 + T-cell immunity [19, 20]. HPV 52 infection was the most common infection among HIV patients receiving antiretroviral therapy [21]. These data support our conclusion. However, these outcomes are contrary to those of Samantha [22] et al., who found that women with HPV18 infection have higher CD4 + T-cell counts in their peripheral blood. Questionably, only 37 samples in the above study particularly limits the credibility of the correlation results. Data from Manaus, Amazonas [23] showed that the HPV genotypes with higher incidence due to reduced CD4 + T cells were 56/59/66 (32.2%), 35/39/68 (28.0%), 52 (21.5%), 16 (19.4%) and 45 (12.9%). That was not identical to HPV18 and HPV52 found in our study. It is possible that the above differences are due to different regions.

In the present study, we found that lower CD4 cell percentages and reduced CD4/CD8 ratios were important determinants of HR-HPV infection, particularly for HPV18 and HPV52. This finding has important implications for vaccination programs. When the CD4 + T-cell percentage or CD4/CD8 ratio is reduced, bivalent and quadrivalent HPV vaccines may not provide effective protection. For this group of people, the nine-valent HPV vaccine should be a better choice.

As cervical cancer screening recommendations have evolved, an increasing number of HPV strains have been found in the early stage of infection. Normal and LSIL account for most HPV-infected women who are initially treated in outpatient departments. These patients have a relatively short duration of viral infection and mild symptoms, and the guidelines recommend follow-up observation without surgery [24]. During this time, a major factor determining viral clearance is the immune status of the body. If HPV clearance could be predicted by monitoring changes in the immune system, it would be of significant clinical importance. With this aim, we constructed PBL prediction models in untreated women.

T-cell percentage and NK cell percentage were identified as independent biomarkers for predicting natural HPV clearance within 12 months. However, there is a nonlinear relationship between CD8 + T cells and HPV clearance, which requires incorporating parity and gravidity into the model to better predict natural HPV clearance. A higher T lymphocyte percentage is associated with a greater likelihood of HPV clearance, while a lower T lymphocyte percentage is associated with a higher likelihood of persistent HPV infection. Conversely, increasing NK cell percentage is associated with a decreased likelihood of HPV clearance and an increased likelihood of persistent HPV infection. Using optimal cutoff values of 67.39% for T-cell percentage, 22.65% for NK cell percentage, and 0.95 for the CD8 + T-cell model risk score, high-risk and low-risk groups can be distinguished for predicting natural HPV clearance.

In the early stages of infection, 90% of HPV can be cleared by the innate immune system alone [25]. NK cells are the main effector cells of the innate immune response and have the ability to recognize and kill virus-infected and transformed cells [26]. Interestingly, although NK cells can kill HPV-infected cells, excess NK cells may not contribute to HPV clearance, perhaps because they have weaker cytotoxic effects. It was reported that cervical tissues with HPV16 infection had more NK cell infiltration than those with HPV18 infection, but these NK cells did not secrete IL-2 and had lower cytotoxicity [27]. Additionally, HPV can reduce the cytotoxicity of NK cells by reducing the expression of NKG2D ligands, which helps the virus evade the surveillance of the host immune system and establish and maintain persistent infections [28].

The results from many studies have agreed that host CD8 + T-cell responses are required to eliminate HPV-infected cells. The regression rate of cervical precancerous lesions is closely related to the presence of intracellular granzyme B-positive cytotoxic T cells [29]. The regression of HPV-induced LSIL requires an effective cell-mediated cytotoxic T-cell response, including antigen-specific CD8 + cytotoxic T cells and CD4 + IL-2/IFNg Th1 helper cells [30]. Peripheral and tumor-infiltrating CD8 + T cells are capable of recognizing epitopes of the HPV-expressed E6/E7 oncogenes. Persistent HPV infection may be associated with the limited ability of CD8 + T cells to generate and present peptide epitopes derived from viral proteins such as E6 and E7[31].

Total T cells also play a central role in the control of viral infections. Wang et al. reported that mouse papillomas were observed only in BALB/c or C57BL/6 mice whose T cells were removed with anti-CD3 antibodies, and they completely regressed within 8 weeks after removal was stopped. In BALB/c or C57BL/6 mice, neither CD4 + nor CD8 + T-cell removal alone was sufficient to form visible papillomas [32]. Mice with UVB-induced systemic immunosuppression were highly susceptible to MmuPV1 infection and eventually developed squamous cell carcinoma [33]. In conclusion, these findings illustrate the critical role of T-cell-mediated immune responses in the clearance of HPV infection by the host.

To the best of our knowledge, our study is the first to discover that T cells, NK cells, and CD8 + T cells can serve as good indicators to predict natural HPV clearance in untreated women (with normal cervix or LSIL) at 12 months. Cutoff values of 67.39%, 22.65%, and 0.95 were identified as the optimal cutoffs for T-cell percentage, NK cell percentage, and CD8 + T-cell model risk score, respectively, demonstrating good performance in distinguishing high-risk and low-risk populations for natural HPV clearance. Some scholars have defined 12 months as a time point, after which patients with HPV infection are considered to have persistent infection, and their cervical lesions are more likely to progress. Our study can help identify this population early and recommend aggressive treatment to facilitate rapid clearance of HPV. However, there are certain limitations to our study. We were unable to perform a detailed and comprehensive assessment of PBL subsets. By detecting PBLs, we are unable to obtain specific information on changes in lymphocyte subtypes and can only obtain macroscopic changes in overall immune function. Furthermore, the sample population included in our follow-up cohort was small, and more multicenter prospective cohort studies are needed in the future to support our findings.

## Conclusion

In conclusion, we found that the percentage of PBL was associated with HPV infection as well as clearance. The lower CD4 + T-cell percentages and CD4/CD8 ratios were important determinants of HR-HPV infection. Low CD4 cell counts often facilitate HPV18 and HPV52 infection, while low CD4/CD8 ratios facilitate HPV 52 infection. People with reduced CD4 + T cells or a decreased CD4/CD8 ratio should not choose the bivalent or quadrivalent HPV vaccine. The nine-valent HPV vaccine could provide better coverage. Moreover, we found that T cells, NK cells, and CD8 + T cells can influence natural HPV clearance. T cells and NK cells can serve as independent biomarkers to predict HPV outcomes. CD8 + T cells need to be adjusted for parity and gravidity to predict natural clearance of HPV. Using a T-cell percentage of 67.39%, an NK cell percentage of 22.65%, and a CD8 + T-cell model risk score of 0.95 as the optimal cutoff value, we can effectively stratify risk populations and identify individuals at high risk for persistent HPV infection. These immunologic parameters will be helpful for patient risk stratification, individualized treatment and follow-up management.

## Electronic supplementary material

Below is the link to the electronic supplementary material.

Additional file 1. Tables S1-S7.

## Data Availability

The analyzed datasets during the present study are available from the corresponding author on reasonable request.

## Abbreviations

HPV: Human papillomavirus
PBL: Peripheral blood lymphocyte
ROC: Time-dependent receiver operating characteristic
AIC: Akaike Information Criterion
C-index: Concordance index
HR-HPV: High-risk HPV
LR-HPV: Low-risk HPV
LSIL: Low-grade squamous intraepithelial lesion
HSIL+: High-grade squamous intraepithelial lesions or worse
HSIL: High-grade squamous intraepithelial lesion
OHR-HPV: Other high-risk HPV
OR: Odd Ratio
HR: Hazard ratio
CI: Confidence interval
AUC: Area under the curve
