# Supplementary material for: Peripheral blood lymphocytes influence human papillomavirus infection and clearance: a retrospective cohort study
Source: Virol J. 2023 May 1;20:80. doi: 10.1186/s12985-023-02039-6 (PMC10152704; doi:10.1186/s12985-023-02039-6)
Supplement: Supplementary file 1 — Additional file 1. Tables S1-S7. [file 12985_2023_2039_MOESM1_ESM.docx]

**Table S1 Comparison of peripheral blood lymphocyte percentage between HPV-infected and noninfected patients**

| PBLs | Median (%) | HPV(-)  n(%) | Any HPV | |  | HR-HPV | |  | LR-HPV | |  | HPV 16 | |  | HPV 18 | |  | HPV 52 | | | HPV 56 | | | | HPV 58 | | | | OHR-HPV | | |
| --- | --- | --- | --- | --- | --- | --- | --- | --- | --- | --- | --- | --- | --- | --- | --- | --- | --- | --- | --- | --- | --- | --- | --- | --- | --- | --- | --- | --- | --- | --- | --- |
|  |  |  | n(%) | *P* |  | n(%) | *P* |  | n(%) | *P* |  | n(%) | *P* |  | n(%) | *P* |  | n(%) | *P* |  | | n(%) | *P* |  | | n(%) | *P* |  | | n(%) | *P* |
| B cells |  |  |  |  |  |  |  |  |  |  |  |  |  |  |  |  |  |  |  |  | |  |  |  | |  |  |  | |  |  |
| ≤12.70 | 12.70 | 142(45.95) | 217(53.32) | 0.05 |  | 183(53.67) | 0.05 |  | 9(52.94) | 0.57 |  | 57(53.77) | 0.16 |  | 19(57.58) | 0.20 |  | 25(54.35) | 0.29 |  | | 9(39.13) | 0.53 |  | | 17(50.00) | 0.65 |  | | 33(61.11) | 0.04* |
| ＞12.70 |  | 167(54.05) | 190(46.68) |  |  | 158(46.33) |  |  | 8(47.06) |  |  | 49(46.23) |  |  | 14(42.42) |  |  | 21(45.65) |  |  | | 14(60.87) |  |  | | 17(50.00) |  |  | | 21(38.89) |  |
| T cells |  |  |  |  |  |  |  |  |  |  |  |  |  |  |  |  |  |  |  |  | |  |  |  | |  |  |  | |  |  |
| ≤70.10 | 70.10 | 150(48.54) | 208(51.11) | 0.50 |  | 175(51.32) | 0.48 |  | 8(47.06) | 0.91 |  | 52(49.06) | 0.93 |  | 19(57.58) | 0.32 |  | 27(58.70) | 0.20 |  | | 12(52.17) | 0.74 |  | | 17(50.00) | 0.87 |  | | 28(51.85) | 0.65 |
| ＞70.10 |  | 159(51.46) | 199(48.89) |  |  | 166(48.68) |  |  | 9(52.94) |  |  | 54(50.94) |  |  | 14(42.42) |  |  | 19(41.30) |  |  | | 11(47.83) |  |  | | 17(50.00) |  |  | | 26(48.15) |  |
| CD4+TCells |  |  |  |  |  |  |  |  |  |  |  |  |  |  |  |  |  |  |  |  | |  |  |  | |  |  |  | |  |  |
| ≤40.30 | 40.30 | 136(44.01) | 223(54.79) | ＜0.01* |  | 194(56.89) | ＜0.01* |  | 8(47.06) | 0.81 |  | 56(52.83) | 0.12 |  | 21(63.64) | 0.03* |  | 34(73.91) | ＜0.01* |  | | 10(43.48) | 0.96 |  | | 17(50.00) | 0.51 |  | | 27(50.00) | 0.41 |
| ＞40.30 |  | 173(55.99) | 184(45.21) |  |  | 147(43.11) |  |  | 9(52.94) |  |  | 50(47.17) |  |  | 12(36.36) |  |  | 12(26.09) |  |  | | 13(56.52) |  |  | | 17(50.00) |  |  | | 27(50.00) |  |
| CD8+TCells |  |  |  |  |  |  |  |  |  |  |  |  |  |  |  |  |  |  |  |  | |  |  |  | |  |  |  | |  |  |
| ≤24.46 | 24.46 | 161(52.10) | 198(48.65) | 0.36 |  | 165(48.39) | 0.34 |  | 7(41.18) | 0.38 |  | 53(50.00) | 0.71 |  | 15(45.45) | 0.47 |  | 21(45.65) | 0.41 |  | | 12(52.17) | 1.00 |  | | 18(52.94) | 0.93 |  | | 27(50.00) | 0.78 |
| ＞24.46 |  | 148(47.90) | 209(51.35) |  |  | 176(51.61) |  |  | 10(58.82) |  |  | 53(50.00) |  |  | 18(54.55) |  |  | 25(54.35) |  |  | | 11(47.83) |  |  | | 16(47.06) |  |  | | 27(50.00) |  |
| NK cells |  |  |  |  |  |  |  |  |  |  |  |  |  |  |  |  |  |  |  |  | |  |  |  | |  |  |  | |  |  |
| ≤14.45 | 14.45 | 160(51.78) | 198(48.65) | 0.41 |  | 162(47.51) | 0.28 |  | 10(58.82) | 0.57 |  | 45(42.45) | 0.10 |  | 15(45.45) | 0.49 |  | 21(45.65) | 0.44 |  | | 15(65.22) | 0.21 |  | | 19(55.88) | 0.65 |  | | 25(46.30) | 0.46 |
| ＞14.45 |  | 149(48.22) | 209(51.35) |  |  | 179(52.49) |  |  | 7(41.18) |  |  | 61(57.55) |  |  | 18(54.55) |  |  | 25(54.35) |  |  | | 8(34.78) |  |  | | 15(44.12) |  |  | | 29(53.70) |  |
| CD4/CD8 |  |  |  |  |  |  |  |  |  |  |  |  |  |  |  |  |  |  |  |  | |  |  |  | |  |  |  | |  |  |
| ≤1.67 | 1.67 | 137(44.34) | 221(54.30) | ＜0.01* |  | 189(55.43) | ＜0.01* |  | 10(58.82) | 0.24 |  | 52(49.06) | 0.40 |  | 20(60.61) | 0.08 |  | 33(71.74) | ＜0.01* |  | | 11(47.83) | 0.75 |  | | 17(50.00) | 0.53 |  | | 27(50.00) | 0.44 |
| ＞1.67 |  | 172(55.66) | 186(45.70) |  |  | 152(44.57) |  |  | 7(41.18) |  |  | 54(50.94) |  |  | 13(39.39) |  |  | 13(28.26) |  |  | | 12(52.17) |  |  | | 17(50.00) |  |  | | 27(50.00) |  |

Median was used as a cutoff

The chi-square test was used to analyze the result (*represents significant differences at p<0.05)

HR-HPV: High-risk HPV including HPV-16, -18, -31, -33, -35, -39, -45, -51, -52, -56, -58, -59, -66, -68

LR-HPV: Low-risk HPV including HPV-6, -11, -42, -43, -81

Other HR-HPV including HPV-31, -33, -35, -39, -45, -51, -59, -66, -68

PBLs: Peripheral blood lymphocytes

**Table S2 Effects of different variables on natural clearance of HPV infection: Bootstrap Analysis**

| **variables** | **HPV Clearance _(Univariate)_** | | |  | **HPV Clearance _(Multivariate)_** | | |
| --- | --- | --- | --- | --- | --- | --- | --- |
|  | **HR_(Unadjusted)_** | **95%CI_(Unadjusted)_** | **C-index** |  | **HR_(Adjusted)_** | **95%CI_(Adjusted)_** | **C-index** |
| Age in years | 1.00 | 0.95-1.04 | 0.55 |  |  |  |  |
| Gravidity | 1.13 | 0.84-1.54 | 0.57 |  |  |  |  |
| Parity | 0.85 | 0.54-1.34 | 0.58 |  |  |  |  |
| Menopause |  |  |  |  |  |  |  |
| No |  |  |  |  |  |  |  |
| Yes | 1.22 | 0.41-Inf | 0.53 |  |  |  |  |
| B Cells | 1.04 | 0.93-1.16 | 0.57 |  | 1.05 | 0.93-1.18 | 0.67 |
| T Cells | 1.09* | 1.02-1.16* | 0.59 |  | 1.09* | 1.01-1.18* | 0.69 |
| CD4+T Cells | 1.04 | 0.97-1.12 | 0.57 |  | 1.04 | 0.96-1.12 | 0.67 |
| CD8+T Cells | 1.05 | 0.99-1.12 | 0.58 |  | 1.09* | 1.01-1.18* | 0.69 |
| NK Cells | 0.91* | 0.84-0.97* | 0.64 |  | 0.88* | 0.81-0.97* | 0.72 |
| CD4/CD8 | 0.85 | 0.38-1.88 | 0.56 |  | 0.73 | 0.28-1.91 | 0.67 |

Calculated by univariate and multivariate Cox regression and bootstrapping

Multivariate Cox regression analysis was adjusted for age, gravidity, parity, and menopause

HR: average hazard ratio calculated across 1,000 iterations

95% CI for HR: average 95% CI for hazard ratio calculated across 1,000 iterations

C-index: average concordance index across 1,000 iterations

* Statistically significant variables: Variables with a mean hazard ratio (HR) and corresponding mean 95% confidence interval (CI) that are entirely between 0 and 1, or greater than 1

**Table S3 Effect of different variables on clearance of HPV Infection in women with HSIL or more severe lesions after treatment: Bootstrap Analysis**

| **variables** | **HPV Clearance _(Univariate)_** | | |  | **HPV Clearance _(Multivariate)_** | | |
| --- | --- | --- | --- | --- | --- | --- | --- |
|  | **HR_(Univariate)_** | **95%CI_(Univariate)_** | **C-index** |  | **HR_(Multivariate)_** | **95%CI_(Multivariate)_** | **C-index** |
| Age in years | 0.98* | 0.95-0.99* | 0.54 |  |  |  |  |
| Gravidity | 0.96 | 0.83-1.11 | 0.53 |  |  |  |  |
| Parity | 0.81* | 0.65-0.99* | 0.57 |  |  |  |  |
| Menopause |  |  |  |  |  |  |  |
| No |  |  |  |  |  |  |  |
| Yes | 0.59 | 0.35-1.01 | 0.55 |  |  |  |  |
| B Cells | 1.04 | 0.99-1.09 | 0.557 |  | 1.04 | 0.99-1.09 | 0.63 |
| T Cells | 0.99 | 0.96-1.02 | 0.53 |  | 0.98 | 0.95-1.01 | 0.62 |
| CD4+T Cells | 0.98 | 0.95-1.02 | 0.53 |  | 1.00 | 0.96-1.05 | 0.62 |
| CD8+T Cells | 0.99 | 0.97-1.02 | 0.52 |  | 0.98 | 0.94-1.01 | 0.63 |
| NK Cells | 1.00 | 0.97-1.03 | 0.53 |  | 1.01 | 0.97-1.04 | 0.61 |
| CD4/CD8 | 0.91 | 0.67-1.22 | 0.54 |  | 1.09 | 0.75-1.58 | 0.62 |

Calculated by univariate and multivariate Cox regression and bootstrapping

Multivariate Cox regression analysis was adjusted for age, gravidity, parity, and menopause

HR: average hazard ratio calculated across 1,000 iterations

95%CI: average 95% CI for hazard ratio calculated across 1,000 iterations

C-index: average concordance index across 1,000 iterations

* Statistically significant variables: Variables with a mean hazard ratio (HR) and corresponding mean 95% confidence interval (CI) that are entirely between 0 and 1, or greater than 1

**Table S4 Risk scores for T-cell prognostic models**

| T-cell percentage (%) | Riskscore | Risk stratification |
| --- | --- | --- |
| 52.48 | 0.237745012 | low |
| 56.19 | 0.319837502 | low |
| 59.58 | 0.419407674 | low |
| 60.4 | 0.447824933 | low |
| 60.93 | 0.467208609 | low |
| 61.37 | 0.483936568 | low |
| 62.03 | 0.510158168 | low |
| 62.37 | 0.524215991 | low |
| 62.78 | 0.541684257 | low |
| 63.11 | 0.556165975 | low |
| 63.47 | 0.572406126 | low |
| 64.31 | 0.612167975 | low |
| 66.18 | 0.710886749 | low |
| 67.14 | 0.767597182 | low |
| 67.39 | 0.783093876 | high |
| 67.67 | 0.800821896 | high |
| 67.83 | 0.811131806 | high |
| 67.92 | 0.81698935 | high |
| 67.95 | 0.81895125 | high |
| 68.2 | 0.83548471 | high |
| 68.24 | 0.838160865 | high |
| 68.29 | 0.841518118 | high |
| 68.69 | 0.868864795 | high |
| 69.29 | 0.911560076 | high |
| 69.31 | 0.913018826 | high |
| 69.39 | 0.918877206 | high |
| 69.78 | 0.947979597 | high |
| 69.87 | 0.95482538 | high |
| 70.59 | 1.011401675 | high |
| 70.68 | 1.018705458 | high |
| 71.24 | 1.065351337 | high |
| 71.36 | 1.075621499 | high |
| 71.53 | 1.090340601 | high |
| 72.03 | 1.134809851 | high |
| 72.08 | 1.13935533 | high |
| 72.09 | 1.140266608 | high |
| 72.57 | 1.184875945 | high |
| 72.69 | 1.196298344 | high |
| 72.74 | 1.201090116 | high |
| 72.9 | 1.216553143 | high |
| 73.18 | 1.244094001 | high |
| 73.51 | 1.277354371 | high |
| 73.83 | 1.310455816 | high |
| 74.43 | 1.374850505 | high |
| 74.44 | 1.375950137 | high |
| 74.72 | 1.407099494 | high |
| 74.72 | 1.407099494 | high |
| 74.79 | 1.414996399 | high |
| 74.86 | 1.422937623 | high |
| 75.08 | 1.448187065 | high |
| 76 | 1.55872228 | high |
| 76.3 | 1.596560171 | high |
| 76.6 | 1.635316574 | high |
| 77.02 | 1.691161196 | high |
| 77.36 | 1.737762518 | high |
| 78.26 | 1.867411669 | high |
| 78.27 | 1.868905261 | high |
| 79.31 | 2.030944169 | high |
| 79.95 | 2.137568067 | high |
| 84.68 | 3.119997072 | high |
| 85.37 | 3.296949127 | high |

**Table S5 Risk scores for NK cell prognostic models**

| NK cell percentage (%) | Riskscore | Risk stratification |
| --- | --- | --- |
| 6.43 | 2.43902329 | high |
| 6.59 | 2.402602989 | high |
| 6.59 | 2.402602989 | high |
| 7.25 | 2.258029096 | high |
| 7.55 | 2.195221799 | high |
| 7.6 | 2.184925092 | high |
| 7.63 | 2.178770265 | high |
| 7.74 | 2.156350529 | high |
| 8.79 | 1.953621451 | high |
| 9.83 | 1.771617011 | high |
| 10.11 | 1.725581423 | high |
| 11.27 | 1.547264041 | high |
| 11.55 | 1.507058279 | high |
| 11.57 | 1.504226742 | high |
| 11.96 | 1.450062897 | high |
| 11.97 | 1.448700031 | high |
| 12.02 | 1.441904891 | high |
| 12.03 | 1.440549692 | high |
| 12.14 | 1.42572631 | high |
| 12.15 | 1.424386318 | high |
| 12.33 | 1.400480664 | high |
| 12.65 | 1.358968056 | high |
| 12.66 | 1.357690807 | high |
| 13.13 | 1.298994898 | high |
| 13.3 | 1.278395225 | high |
| 13.52 | 1.252220992 | high |
| 13.87 | 1.211680165 | high |
| 14.72 | 1.118604269 | high |
| 14.95 | 1.094671828 | high |
| 15.07 | 1.082389301 | high |
| 15.42 | 1.047346798 | high |
| 15.47 | 1.042434209 | high |
| 15.68 | 1.022051702 | high |
| 16.02 | 0.989893005 | high |
| 16.03 | 0.988962637 | high |
| 16.2 | 0.973279507 | high |
| 16.8 | 0.919888787 | high |
| 17.01 | 0.901902387 | high |
| 17.58 | 0.854835196 | high |
| 17.98 | 0.823279989 | high |
| 18.06 | 0.817110125 | high |
| 18.19 | 0.807182561 | high |
| 18.25 | 0.802641378 | high |
| 18.36 | 0.794382128 | high |
| 18.39 | 0.792144393 | high |
| 19.26 | 0.729921379 | high |
| 19.26 | 0.729921379 | high |
| 19.59 | 0.707619585 | high |
| 19.75 | 0.697053176 | high |
| 20.29 | 0.662542713 | high |
| 20.66 | 0.639888284 | high |
| 22.65 | 0.530686878 | high |
| 23.31 | 0.498753401 | low |
| 23.52 | 0.489001376 | low |
| 24.16 | 0.460441377 | low |
| 27.37 | 0.340476612 | low |
| 27.58 | 0.333819342 | low |
| 27.61 | 0.332878989 | low |
| 28.5 | 0.306155012 | low |
| 29.52 | 0.27815543 | low |
| 33.19 | 0.196976956 | low |

**Table S6 Risk scores for CD8+ T-cell prognostic models**

| CD8+T-cell percentage (%) | Gravity | Parity | Riskscore | Risk stratification |
| --- | --- | --- | --- | --- |
| 12.8 | 1 | 1 | 0.29995 | low |
| 16.22 | 6 | 3 | 0.738048 | low |
| 16.49 | 2 | 1 | 0.509317 | low |
| 16.71 | 8 | 2 | 2.053082 | high |
| 17.46 | 4 | 3 | 0.44107 | low |
| 18.93 | 4 | 2 | 0.721315 | low |
| 19.29 | 4 | 1 | 1.0998 | high |
| 19.57 | 4 | 3 | 0.503906 | low |
| 19.86 | 4 | 2 | 0.764926 | low |
| 20.47 | 0 | 0 | 0.539315 | low |
| 20.53 | 5 | 2 | 1.073422 | high |
| 20.54 | 3 | 3 | 0.39825 | low |
| 20.56 | 2 | 2 | 0.441814 | low |
| 21.25 | 5 | 0 | 2.495444 | high |
| 21.45 | 3 | 1 | 0.937004 | low |
| 21.67 | 5 | 3 | 0.77393 | low |
| 21.75 | 3 | 1 | 0.954916 | low |
| 21.85 | 3 | 0 | 1.432275 | high |
| 21.86 | 3 | 2 | 0.64515 | low |
| 22.29 | 1 | 1 | 0.546008 | low |
| 22.35 | 4 | 2 | 0.895113 | low |
| 22.38 | 5 | 4 | 0.543057 | low |
| 22.54 | 1 | 1 | 0.554693 | low |
| 23.53 | 4 | 2 | 0.964329 | high |
| 23.88 | 2 | 1 | 0.812027 | low |
| 23.89 | 5 | 1 | 1.977874 | high |
| 24.03 | 2 | 2 | 0.55 | low |
| 24.26 | 3 | 1 | 1.118851 | high |
| 24.45 | 1 | 1 | 0.625765 | low |
| 24.48 | 1 | 1 | 0.626951 | low |
| 24.74 | 1 | 0 | 0.949907 | high |
| 24.81 | 1 | 1 | 0.640148 | low |
| 24.86 | 3 | 2 | 0.779651 | low |
| 24.92 | 4 | 1 | 1.569096 | high |
| 26.26 | 3 | 2 | 0.851684 | low |
| 26.38 | 3 | 1 | 1.279051 | high |
| 26.54 | 4 | 2 | 1.166108 | high |
| 26.63 | 4 | 3 | 0.786839 | low |
| 27.22 | 3 | 2 | 0.904889 | low |
| 27.68 | 5 | 1 | 2.512432 | high |
| 27.83 | 2 | 1 | 1.041963 | high |
| 28.67 | 3 | 2 | 0.991617 | high |
| 29.87 | 3 | 2 | 1.069646 | high |
| 30.48 | 2 | 2 | 0.826375 | low |
| 31.11 | 4 | 1 | 2.319191 | high |
| 31.43 | 3 | 2 | 1.180332 | high |
| 31.76 | 3 | 2 | 1.205176 | high |
| 32.06 | 4 | 2 | 1.652187 | high |
| 32.47 | 4 | 2 | 1.695503 | high |
| 33.21 | 0 | 0 | 1.205274 | high |
| 34.36 | 2 | 2 | 1.055699 | high |
| 34.76 | 3 | 1 | 2.17075 | high |
| 35.17 | 4 | 3 | 1.348944 | high |
| 36.17 | 0 | 0 | 1.452876 | high |
| 36.18 | 3 | 3 | 1.068801 | high |
| 36.64 | 3 | 2 | 1.639935 | high |
| 36.83 | 0 | 0 | 1.514681 | high |
| 36.98 | 2 | 2 | 1.245554 | high |
| 39.1 | 1 | 0 | 2.351438 | high |
| 39.47 | 2 | 2 | 1.457542 | high |
| 41.71 | 5 | 2 | 4.08677 | high |

**Table S7. Baseline characteristics of patients involved in follow-up and those not.**

| Variable | Patients not involved in follow-up(n=243) | Patients involved in follow-up(n=164) | *P* |
| --- | --- | --- | --- |
| Age |  |  |  |
| ≤46 | 127/243 (52.26) | 108/164 (65.85) | 0.01^*a^ |
| ＞46 | 116/243 (47.74) | 56/164 (34.15) |  |
| Gravidity |  |  |  |
| ≤3 | 146/241 (60.08) | 102/164 (62.20) | 0.74^a^ |
| ＞3 | 95/241 (39.42) | 62/164 (37.80) |  |
| Parity |  |  |  |
| ≤2 | 176/242(72.73) | 130/164(79.27) | 0.13^a^ |
| ＞2 | 66/242(27.27) | 34/164 (20.73) |  |
| Menopause |  |  |  |
| Yes | 73/243 (30.04) | 39/164(23.78) | 0.17^a^ |
| No | 170/243 (69.96) | 125/164 (76.22) |  |
| Cervical Lesions |  |  |  |
| Normal/LSIL | 85/243(34.9 8) | 61/164(37.20) | 0.65^a^ |
| HSIL+ | 158/243(65.02) | 103/164(62.80) |  |
| B cells | 12.50(9.80-15.40) | 11.89(9.58-16.28) | 0.88^b^ |
| T cells | 69.63(8.57) | 69.40(7.13) | 0.78^c^ |
| CD4+T cells | 39.34(7.94) | 39.51(5.80) | 0.80^c^ |
| CD8+T cells | 24.90(20.50-30.10) | 24.33(20.53-29.72) | 0.51^b^ |
| NK cells | 14.23(10.04-20.89) | 15.42(11.91-20.04) | 0.21^b^ |
| CD4/CD8 | 1.57(1.21-2.08) | 1.61(1.19-2.12) | 0.74^b^ |
| HPV |  |  |  |
| Single | 178/243(73.25) | 117/164(71.34) | 0.67^a^ |
| Multiple | 65/243(26.75) | 47/164(28.66) |  |
| HR-HPV |  |  |  |
| Negative | 13/243(5.35) | 5/164(3.05) | 0.27^a^ |
| Positive | 230/243(94.65) | 159/164(96.95) |  |
| HPV16 |  |  |  |
| Negative | 148/243(60.91) | 98/164(59.76) | 0.82^a^ |
| Positive | 95/243(39.09) | 66/164(40.24) |  |
| HPV18 |  |  |  |
| Negative | 211/243(86.83) | 146/164(89.02) | 0.51^a^ |
| Positive | 32/243(13.17) | 18/164(10.98) |  |
| HPV52 |  |  |  |
| Negative | 201/243(82.72) | 134/164(81.71) | 0.79^a^ |
| Positive | 42/243(17.28) | 30/164(18.29) |  |
| HPV56 |  |  |  |
| Negative | 226/243(93.00) | 158/164(96.34) | 0.15^a^ |
| Positive | 17/243(7.00) | 6/164(3.66) |  |
| HPV58 |  |  |  |
| Negative | 213/243(87.65) | 141/164(85.98) | 0.62^a^ |
| Positive | 30/243(12.35) | 23/164(14.02) |  |
| OHR-HPV |  |  |  |
| Negative | 179/243(73.66) | 121/164(73.78) | 0.98^a^ |
| Positive | 64/243(26.34) | 43/164(26.22) |  |

HR-HPV: High-risk HPV including HPV-16, -18, -31, -33, -35, -39, -45, -51, -52, -56, -58, -59, -66, -68; LR-HPV: OHR-HPV: Other High-Risk HPV including HPV-31, -33, -35, -39, -45, -51, -59, -66, -68; ^a^ was calculated by chi-square test; ^b^ was calculated by nonparametric test of Mann–Whitney U; ^c^ was calculated by independent samples t -test; ^*^ statistically significant at p<0.05.
